# Crack Size Identification for Bearings Using an Adaptive Digital Twin

**DOI:** 10.3390/s21155009

**Published:** 2021-07-23

**Authors:** Farzin Piltan, Jong-Myon Kim

**Affiliations:** Department of Electrical, Electronics and Computer Engineering, University of Ulsan, Ulsan 44610, Korea; piltanfarzin@gmail.com

**Keywords:** rotating machine, bearing, digital twin, gaussian process regression, Laguerre filter, fuzzy logic, proportional integral observer, Lyapunov robust algorithm, adaptive technique, support vector machine, fault diagnosis

## Abstract

In this research, the aim is to investigate an adaptive digital twin algorithm for fault diagnosis and crack size identification in bearings. The main contribution of this research is to design an adaptive digital twin (ADT). The design of the ADT technique is based on two principles: normal signal modeling and estimation of signals. A combination of mathematical and data-driven techniques will be used to model the normal vibration signal. Therefore, in the first step, the normal vibration signal is modeled to increase the reliability of the modeling algorithm in the ADT. Then, to help challenge the complexity and uncertainty, the data-driven method will solve the problems of the mathematically based algorithm. Thus, first, Gaussian process regression is selected, and then, in two steps, we improve its resistance and accuracy by a Laguerre filter and fuzzy logic algorithm. After modeling the vibration signal, the second step is to design the data estimation for ADT. These signals are estimated by an adaptive observer. Therefore, a proportional-integral observer is then combined with the proposed technique for signal modeling. Then, in two stages, its robustness and reliability are strengthened using the Lyapunov-based algorithm and adaptive technique, respectively. After designing the ADT, the residual signals that are the difference between original and estimated signals are obtained. After that, the residual signals are resampled, and the root means square (RMS) signals are extracted from the residual signals. A support vector machine (SVM) is recommended for fault classification and crack size identification. The strength of the proposed technique is tested using the Case Western Reserve University Bearing Dataset (CWRUBD) under diverse torque loads, various motor speeds, and different crack sizes. In terms of fault diagnosis, the average detection accuracy in the proposed scheme is 95.75%. In terms of crack size identification for the roller, inner, and outer faults, the proposed scheme has average detection accuracies of 97.33%, 98.33%, and 98.33%, respectively.

## 1. Introduction

Bearings are components that are used in various industries from boiler feed pumps to automotive transmissions to reduce friction. Due to the many applications of these components, different research has been presented on how to evaluate the associated condition monitoring processes. However, the common denominator in all of these studies is the complexity of the bearings and their nonlinear behavior. Therefore, in this study, the aim is to provide a robust and reliable method for fault diagnosis and crack size identification in bearings in an active machine [1].

Generally, there are four types of defined bearing faults, which are the roller fault, inner fault, outer fault, and cage fault. These faults cause different behaviors, and consequently, they have different signal profiles. To diagnose faults in bearings, first, data collection must be performed [2]. For condition monitoring, depending on the type of work or project, vibration sensors, acoustic emission sensors, or current sensors can be used. In this work, vibration signals are used for bearing state detection and diagnosis [3]. 

Different techniques, such as data-driven algorithms [3] and model-based methods [4], have been introduced to diagnose faults in bearings. The high resolution the optical flow approach with total generalized variation (TGV)-based optical approach for crack detection is introduced in [5,6]. The most important challenges of model-based methods are the increased complexity and decreased modeling accuracy in unknown conditions. The combination of ensemble empirical mode decomposition (EEMD), correlation coefficient (CC), and singular value decomposition (SVD) technique is introduced in [7]. The combination of convolutional neural network (CNN) model and a deep forest (gcForest) model is used to fault diagnosis of bearing and proposed in [8]. On the other hand, data-driven techniques have the challenges of unreliability, especially for accurately characterizing nonlinear and non-stationary signals, as well as high dependences on the type and accuracy of the data [9]. Therefore, the proposed method in this research is a combination between a model-based approach and a data-driven algorithm.

In designing model-based methods, signal estimation is of great importance. To estimate the signals, the first step is to model the system. Various linear and nonlinear techniques for systems modeling have been introduced that can be divided into two categories: mathematically based modeling and modeling based on system identification techniques [3,10]. Mathematically based techniques, based on extracting the dynamic relationships between different parts of the system, are able to extract the dynamic equations of the system. The complexity of the modeling, especially in nonlinear systems, and the reduction of accuracy in unknown conditions are the most important challenges of the modeling-based technique [10]. Moreover, the multi-dimensional mathematical models can be used for evaluating the impact of selected factors on the vibration rolling ball bearings [11]. System identification-based methods are very close to data-driven algorithms. In these methods, the system behavior is modeled according to the signals extracted from the systems in different conditions [12]. Different methods, such as autoregressive [12], autoregressive with external inputs [12], Gaussian process regression [13], neuro-fuzzy [14], nonlinear autoregressive [15], and neural networks [16], are among the methods of system identification. The main challenge in all of these methods is their reliability due to the strong dependence on data [12]. In this research, the focus is on the simultaneous combination of mathematically based and system identification methods for bearing modeling.

The second part is designing model-based approaches for the signal estimation. Estimators are used to enhance the power of modeling unknown conditions and more accurately detect the system performance in different conditions [3]. Although different estimation techniques have been used in research articles, they can be divided into two main groups: linear estimation and nonlinear estimation techniques [17]. Linear estimation techniques, such as Proportional Integral (PI) [12] and Proportional Multi-Integral observers [18], can provide a good response in less-complex systems. The most important positive point of linear estimators is their simple implementation. Estimation accuracy and robustness are introduced as the most important limitations of linear observers [18]. However, in complex and nonlinear systems, nonlinear estimation techniques, such as feedback linearization [17], sliding mode [10], backstepping [19], Lyapunov-based [20], fuzzy [21], and neural network [22] observers, are suggested. High accuracy and robustness can be the most important positive attributes of the nonlinear compensators [22]. However, the complexity of designing these techniques is recognized as their most important limitation [4,10]. The digital twins are a relatively novel way to model physical systems. In these methods, physical systems are reliably modeled, and even the system’s model can be used to generate data. Digital twin technology is becoming more popular. The digital twins are anticipated to grow by about 38% and reach $16 billion by 2023. Digital twins are changing the style of work is achieved in various industries with diverse applications such as manufacturing, healthcare, supply chain, predictive maintenance, automotive, self-driving car development, and retail [23]. Therefore, in this research, the linear estimation technique is used, which has its robustness and accuracy improved by the application of nonlinear and intelligent techniques. Thus, a combination of the proposed modeling and estimation algorithms is used to design a proposed digital twin for the fault diagnosis of bearings. 

After processing data using the model-based or/and data-driven approaches, the next step is to use classical [7,17] or learning algorithms [24,25] to perform signal classification. Classical techniques, such as the sliding mode method, have been used in various articles to determine the best value for the threshold employed in this method [10,17]. Similarly, deep learning [24] and machine learning approaches [25] have been widely used in various research works to perform state classification. In this work, a machine learning-based technique, a support vector machine (SVM) [26], is used for signal classification.

This research makes the following contributions:

- The first contribution is about bearing vibration signal modeling. The combination of mathematical vibration bearing signal modeling, Gaussian Process Regression (GPR), input-output Laguerre filter, and fuzzy approach, MGPRLF, is used for bearing vibration signal modeling.
- The second contribution is proposed to adaptive digital twin. A combination of MGPRLF and proposed observer (hence is a combination of PI observer, Lyapunov robust technique, and adaptive fuzzy algorithm) is recommended to design proposed adaptive digital twin. This proposed technique is suggested to prepare the vibration signals for easier and higher-accuracy classification.
- A combination of the resulting adaptive digital twin and a machine learning (SVM) algorithm is recommended for signal classification and crack size identification.

This research article has the following parts. The dataset is described in Section 2. The proposed scheme, which includes the adaptive digital twin, residual signal computation, and signal classification, is described in Section 3. The results are analyzed and discussed in the Section 4. The conclusion is presented and explained in Section 5. 

## 2. Dataset

**Figure 1:**
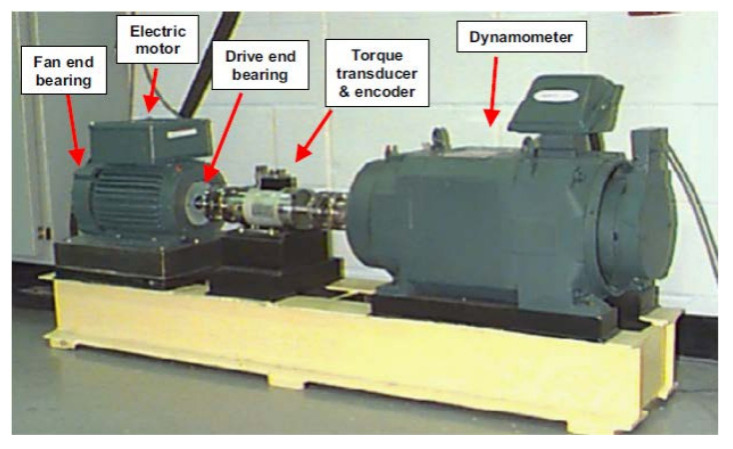
The testbed of CWRUBD to collect the data [27].

To test the power of the proposed adaptive digital twin algorithm, Bearing Case Western Reverse University Bearing Dataset (CWRUBD) is suggested in this work. Figure 1 illustrates the experimental setup for CWRUBD. 

In the CWRUBD, an electric motor with 2-horsepower (hp) is used. This motor is used to simulant a shaft that a transducer and encoder are mounted. The electric torque is transferred from the shaft to the control system using a dynamometer. This electric motor is provided with 4-different speeds to rotate the roller bearings 6205-2RS JEM SKF including 1797-rotation per minute (RPM), 1772-RPM, 1750-RPM, and 1730-RPM [27,28]. To collect the data, the vibration sensor is suggested. Vibration data were collected using accelerometers that were placed at the 12 o’clock position at both the drive end and fan end of the motor housing. The 16-channel data acquisition module is used to collect the data and transfer them to the MATLAB environment. The vibration signals were collected via installed-on-bearing housing. Single-point faults with three different crack sizes (i.e., severity levels) of 0.007, 0.014, and 0.021 inches in diameter were seeded on the drive-end bearings at different bearing locations as the outer fault (OF), inner fault (IF), and the roller fault (RF), respectively. Data were collected for the three fault conditions and bearings in normal conditions (NC). The data were recorded at a 48 kHz sampling rate under four different motor loads from 0 to 3 hp. The basic information about the CWRUBD is listed in Table 1 [27,28].

Furthermore, Table 2 shows the CWRUBD signal condition test information.

## 3. Proposed Scheme

**Figure 2:**
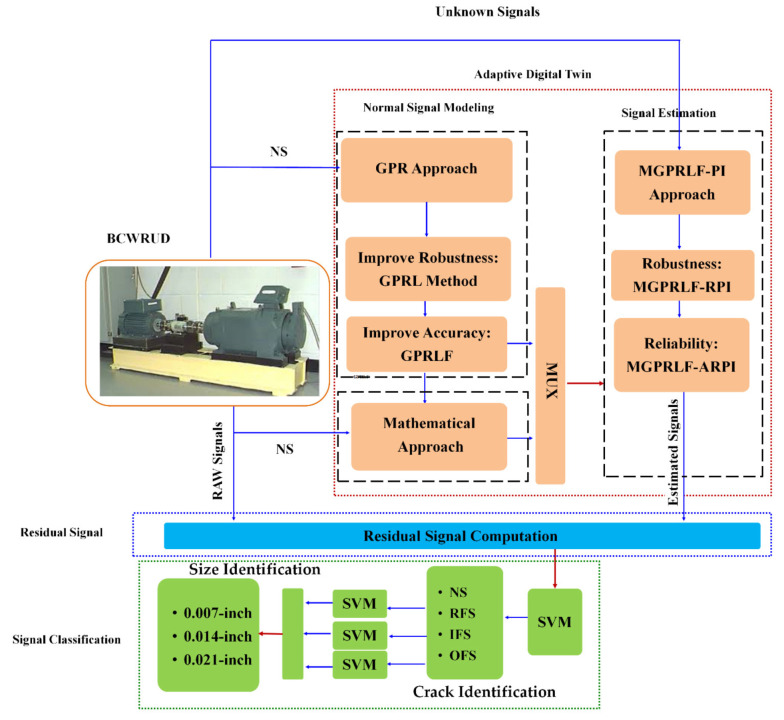
The combination of proposed adaptive digital twin and machine learning for the bearing crack detection and size identification.

Figure 2 illustrates the block diagram of the proposed algorithm. The proposed scheme has three main parts: (a) an adaptive digital twin to model and estimate the bearing signals, (b) residual signal computation to evaluate the new feature for signal classification, and (c) crack detection and size identification using SVM.

The adaptive digital twin (ADT) has two main parts: normal signal modeling and estimation units. The adaptive digital twin is suggested for bearing signal modeling and estimation. 

To estimate the bearing signals using ADT, the first step is vibration bearing signal modeling in normal conditions. The bearing signal modeling is used to extract the state-space function. To extract the state-space function from the bearing vibration signal, two techniques are used in parallel: the signal identification part and the mathematical vibration bearing signal modeling section. First, the vibration bearing signal in normal conditions is modeled using the mathematical-based vibration signal technique. However, this technique is reliable in certain conditions, but it has limitations in uncertainties (e.g., variation in motor torque load and motor speed). To address this issue, the signal identification technique is recommended. The Gaussian Process Regression (GPR) technique is a data-driven algorithm for function approximation. The GPR algorithm is a nonlinear regression technique used to model nonlinear and non-stationary signals using nonlinear functions (kernels), but it is not accurate and reliable for vibration signal modeling. Thus, to increase the robustness, the GPR technique is integrated with an input-output Laguerre filter, which, from now on, is called the GPRL. The state estimation function in GPR technique is filtered using the feedback of the state of the bearing signal modeling to the modeled signal. Next, to improve the accuracy of the signal modeling, the combination of the GPRL and two inputs (error and integral of error) fuzzy technique, hence called the GPRLF, is suggested. The fuzzy algorithm is a rule-based nonlinear technique that can be used for modeling, control, and prediction.

Regarding the fuzzy algorithm, the fuzzy technique tries to reduce the error and integral of error of the system/signal modeling. It means that the fuzzy algorithm is used to improve the accuracy and flexibility (rule-based technique) of the system/signal modeling. Finally, the mathematical vibration signal modeling is integrated with the GPRLF to form the MGPRLF. Therefore, the normal signal (e.g., when the torque load is 0-hp) is selected for tuning the signal modeling.

After modeling and extracting the state-space equation from the normal signal, the proposed observer is suggested for vibration signal estimation and reduce the effect of uncertainties. Therefore, first, the Proportional Integral (PI) observer that is modeled by the MGPRLF, hence called the MGPRLF-PI, is proposed. In this technique, the integral function is selected to reducing the effect of the unknown condition in the vibration signal. Apart from the simplicity of the MGPRLF-PI technique, robustness and reliability are two main limitations of this approach. The Lyapunov technique is integrated with the MGPRLF-PI observer to improve the robustness (hence is called MGPRLF-RPI). In this approach, the Lyapunov technique is selected to improve the robustness of signal estimation against the unknown condition in the vibration signal. Next, to increase the reliability of the MGPRLF-RPI, the adaptive approach is integrated with the MGPRLF-RPI algorithm, which, from now on, is called MGPRLF-ARPI (ADT). The adaptive approach is used to auto-tune the coefficient to estimate the unknown condition. In this step, the normal signal (e.g., when the torque load is 0-hp) is selected to tuning the estimator. The other signals in normal and abnormal conditions are used as real-time vibration data and used for tests. It is clear that, in normal conditions, the power of signal estimation is better than the others.

After designing an adaptive digital twin using the proposed MGPRLF-ARPI (ADT) technique, the residual signal that is a difference between the original bearing raw signals and estimated signals using the ADT is computed.

Finally, a support vector machine (SVM) is suggested for fault pattern identification and crack size identification. Thus, the combination of the ADT and SVM is suggested for fault pattern recognition and crack size identification. 

### 3.1. Adaptive Digital Twin

Figure 2 describes using GRP to design the ADT. The GPR technique is a nonlinear regression technique used to model nonlinear and non-stationary signals using nonlinear functions (kernels). The state-space of the GPR algorithm is introduced by the following definition [29].

(1) $$\{\begin{array}{l} X_{GPR}\left(k+1\right)=\left[{\mathbb{C}}_{GPR}X_{GPR}\left(k\right)+\delta_{i}X_{i}\left(k\right)\right]+e_{GPR}\left(k\right)\\ Y_{GPR}\left(k\right)={\left(\delta_{o}\right)}^{T}\left(x_{n}\right){\mathbb{C}}_{GPR}{}^{-1}\times X_{GPR}\left(k\right) \end{array}.$$

Here, $X_{GPR}\left(k\right),\;X_{i}\left(k\right),\;e_{GPR}\left(k\right),\;Y_{GPR}\left(k\right),\;{\mathbb{C}}_{GPR},$ and $(\delta_{i},\;\left(\left(\delta_{o}{)}^{T}\left(x_{n}\right)\right)\right)$ are the state of the bearing signal modeling using the GPR technique, the measurable vibration signal, the error of signal modeling using the GPR algorithm, the signal modeled by the GPR technique, the covariance matrix using the GPR technique, and the coefficient of signal modeling using the GPR algorithm, respectively. The covariance matrix, ${\mathbb{C}}_{GPR},$, is represented in the following definition.

(2) $${\mathbb{C}}_{GPR}=\alpha^{2}e^{\left(-0.5X_{GPR}{}^{T}H^{-1}X_{GPR}\right)}+\varepsilon .$$

(3) $$H=diag{\left(k\right)}^{2}$$

Here, α, ε, and k, respectively, correspond to the signal variance, noise variance, and the kernel width.

(4) $$\delta_{o}\left(x_{n}\right)=\mathbb{C}{\left(x_{n},X_{i}\right)}^{T}$$

Error of signal modeling using the GPR algorithm, $e_{GPR}\left(k\right)$, is represented as the following equation.

(5) $$e_{GPR}\left(k\right)=Y_{GPR}\left(k\right)-Y_{GPR}\left(k-1\right)$$

However, the GPR algorithm is a nonlinear regression technique used to model nonlinear and non-stationary signals; it is not accurate and reliable for vibration signal modeling. To improve the robustness, the GPR method is integrated with the Laguerre filter, which, from now on, is called the GPRL. Thus, the combination of the GPR and Laguerre filter can be represented by the following definition.

(6) $$\{\begin{array}{l} X_{GPRL}\left(k+1\right)=\left[{\mathbb{C}}_{GPRL}X_{GPRL}\left(k\right)+\delta_{i}X_{i}\left(k\right)+\delta_{o}Y_{GPRL}\left(k\right)\right]+e_{GPRL}\left(k\right)\\ Y_{GPRL}\left(k\right)={\left(\delta_{o}\right)}^{T}\left(x_{n}\right){\mathbb{C}}_{GPR}{}^{-1}\times X_{GPRL}\left(k\right) \end{array},$$

Here, $X_{GPRL}\left(k\right),e_{GPRL}\left(k\right),Y_{GPRL}\left(k\right),$ and ${\mathbb{C}}_{GPRL}\;$ are the state of the bearing signal modeling using the GPRL technique, the error of signal modeling using the GPRL algorithm, the modeled signal by the GPRL method, and the covariance matrix using the GPRL algorithm, respectively. According to (6), the state estimation function in GPR technique is filtered using the feedback of the state of the bearing signal modeling to the modeled signal.

(7) $${\mathbb{C}}_{GPRL}=\alpha^{2}e^{\left(-0.5X_{GPRL}{}^{T}H^{-1}X_{GPRL}\right)}+\varepsilon$$

Moreover, the error of signal modeling using the GPRL algorithm, $e_{GPRL}\left(k\right)$, is represented as the following equation.

(8) $$e_{GPRL}\left(k\right)=Y_{GPRL}\left(k\right)-Y_{GPRL}\left(k-1\right)$$

Next, to improve the accuracy of the signal modeling, the combination of the GPRL and fuzzy technique, hence called the GPRLF, is suggested. The fuzzy algorithm is a rule-based nonlinear technique that can be used for modeling, control, and prediction. To design and implement the fuzzy algorithm, the following steps are used. 

Inputs/outputs: The Proportional Integral fuzzy-like technique is recommended in this work.

Linguistic variables/Rule base/Membership function: Three linguistic variables are recommended for inputs and output. Moreover, the AND operator is used in the input and nine rule-bases are defined. The triangular membership function is suggested in this work.

Fuzzy Inference Engine (FIE): The Mamdani FIM is used for modeling the vibration signal. 

Aggregation: The Max-Min aggregation technique is used for the vibration signal modeling.

Defuzzification: The last step is defuzzification. The Center of Gravity (CoG) technique is recommended for defuzzification in this work. Regarding the fuzzy algorithm, the fuzzy technique tries to reduce the error and integral of error of the system/signal modeling. It means that the fuzzy algorithm is used to improve the accuracy and flexibility (rule-based technique) of the system/signal modeling.

Thus, the combination of the GPRL and fuzzy logic algorithm (GPRLF) is represented by the following definition.

(9) $$\{\begin{array}{l} X_{GPRLF}\left(k+1\right)=\left[{\mathbb{C}}_{GPRLF}X_{GPRLF}\left(k\right)+\delta_{i}X_{i}\left(k\right)+\delta_{o}Y_{GPRLF}\left(k\right)+\delta_{f}Y_{f}\left(k\right)\right]+e_{GPRLF}\left(k\right)\\ Y_{GPRLF}\left(k\right)={\left(\delta_{o}\right)}^{T}\left(x_{n}\right){\mathbb{C}}_{GPRLF}{}^{-1}\times X_{GPRLF}\left(k\right) \end{array},$$

Here, $X_{GPRLF}\left(k\right),e_{GPRLF}\left(k\right),Y_{GPRLF}\left(k\right),Y_{f}\left(k\right)$,$\;{\mathbb{C}}_{GPRLF},$ and $\delta_{f}$ are the state of the bearing signal modeling using the GPRLF technique, the error of signal modeling using the GPRLF algorithm, the modeled signal by the GPRLF method, the modeled signal using the fuzzy algorithm to improve the accuracy and flexibility, the covariance matrix using the GPRLF algorithm, and the coefficient of the modeled signal using the fuzzy algorithm, respectively.

(10) $${\mathbb{C}}_{GPRLF}=\alpha^{2}e^{\left(-0.5X_{GPRLF}{}^{T}H^{-1}X_{GPRLF}\right)}+\varepsilon$$

Furthermore, the error of signal modeling using the GPRLF algorithm, $e_{GPRLF}\left(k\right)$, is represented as the following equation.

(11) $$e_{GPRLF}\left(k\right)=Y_{GPRLF}\left(k\right)-Y_{GPRLF}\left(k-1\right)$$

After modeling the normal vibration signal using the data-driven GPRLF algorithm, mathematical signal modeling is recommended to increase the reliability. Thus, the mathematical technique for modeling the vibration bearing signal is represented as the following equation.

(12) $$F_{D\left(q\right)}={\mathbb{Z}}_{D\left(q\right)}\left[\ddot{q}\right]+{\mathbb{N}}_{D}\left(q,\dot{q}\right)+\theta_{D}$$

where $F_{D\left(q\right)},{\mathbb{Z}}_{D\left(q\right)},\ddot{q},{\mathbb{N}}_{D}\left(q,\dot{q}\right),$ and $\theta_{D}$ are the external source forces, the mass of bearing matrices, the acceleration vibration signal that is measured by a vibration sensor, a nonlinear term for modeling the bearing, and the unknown condition (hence called uncertainty), respectively. The uncertainty can be modeled using the following definition.

(13) $$\theta_{D}=\theta_{RF}+\theta_{IF}+\theta_{OF}$$

Here, $\theta_{RF},\theta_{IF},$ and $\theta_{OF}\;$ are the effect of the roller fault, the effect of the inner fault, and the effect of the outer fault, respectively. Moreover, the effect of the roller fault, $\theta_{RF}$, is represented as the following equation.

(14) $$\theta_{RF}=Max(\theta_{IOF}Cos\left(\theta_{u}\right)+\theta_{IOF}Sin\left(\theta_{u}\right)-\varphi_{\alpha }-\theta_{f}$$

Furthermore, the effect of the inner fault, $\;\theta_{RF}$, and outer fault, $\theta_{OF}$, are represented as the following equations, respectively.

(15) $$\theta_{IF}=Max(\theta_{IOF}Cos\left(\theta_{u}\right)-\theta_{IOF}Sin\left(\theta_{u}\right)-2\left(\varphi_{\alpha }-\theta_{f}\right)$$

(16) $$\theta_{OF}=Max(\theta_{IOF}Cos\left(\theta_{u}\right)+1.5\left(\theta_{IOF}Sin\left(\theta_{u}\right)\right)-\varphi_{\alpha }+\theta_{f}$$

and

(17) $$\theta_{IOF}=\theta_{IF}-\theta_{OF}$$

(18) $$\theta_{u}=\frac{2\pi \left(j-1\right)}{N_{RC}}+\varphi_{\alpha }+\theta_{OF}$$

Here, $\varphi_{\alpha },N_{RC}$, and $\theta_{f}$ are the angular velocity of rotor, the number of rollers in the bearing, and the difference between two reference angular positions, respectively. Thus, the state-space definition for the mathematical modeling of the bearing is introduced using the following equation.

(19) $$\{\begin{array}{l} X_{M}\left(k+1\right)=\delta_{XD}\left(X_{D}\left(k\right),X_{Di}\left(k\right)\right)+\chi_{XD}\left(X_{D}\left(k\right),X_{Di}\left(k\right)\right)\\ Y_{M}\left(k\right)={\left(\delta_{YD}\right)}^{T}X_{M}\left(k\right) \end{array},$$

Here, $\delta_{XD}\left(X_{D}\left(k\right),X_{Di}\left(k\right)\right),\chi_{XD}\left(X_{D}\left(k\right),X_{Di}\left(k\right)\right),X_{M},Y_{M}\left(k\right),$ and $\delta_{YD}\;$ are the nonlinear term of the bearing using mathematically-based vibration modeling, the uncertainty term of the bearing using mathematically-based vibration modeling, the state of the vibration signal modeling using the mathematical approach, the modeled vibration signal using the mathematical technique, and the coefficient, respectively. Thus, based on Equations (9) and (19), the proposed MGPRLF technique is represented as the following equation.

(20) $$\{\begin{array}{l} X_{MGPRLF}\left(k+1\right)=X_{M}\left(k\right)+X_{GPRLF}\left(k\right)\\ Y_{MGPRLF}\left(k\right)=Y_{M}\left(k\right)+Y_{GPRLF}\left(k\right) \end{array}.$$

Here, $X_{GPRLF}\left(k\right)$ and $Y_{GPRLF}\left(k\right)$ are the state of the bearing signal modeling using the MGPRLF technique and the modeled signal by the GPRLF method, respectively. After modeling and extracting the state-space equation from the normal signal, the proposed observer is suggested for vibration signal estimation and reduce the effect of uncertainties. Thus, the PI observer procedure is recommended for signal estimation. Thus, the PI observer that is modeled by MGPRLF, MGPRLF-PI, is represented by the following definition:

(21) $$\{\begin{array}{c} X_{MGPRLF-PI}\left(k+1\right)=[{\mathbb{C}}_{GPRLF}X_{MGPRLF-PI}\left(k\right)+\delta_{i}X_{i}\left(k\right)+\delta_{o}Y_{MGPRLF-PI}\left(k\right)\\ +\delta_{f}Y_{f}\left(k\right)]+e_{GPRLF}\left(k\right)+\phi_{MGPRLF-PI}\\ Y_{MGPRLF-PI}\left(k\right)={\left(\delta_{o}\right)}^{T}\left(x_{n}\right){\mathbb{C}}_{GPRLF}{}^{-1}\times X_{MGPRLF-PI}\left(k\right) \end{array},$$

Here, $X_{MGPRLF-PI}\left(k\right),Y_{MGPRLF-PI}\left(k\right),$ and $\phi_{MGPRLF-PI}$ are the state of the bearing signal estimation using the MGPRLF-PI technique, the estimated signal by the MGPRLF-PI method, and the uncertainty estimation using the MGPRLF-PI algorithm, respectively. In this technique, the integral function is selected to reducing the effect of the unknown condition in the vibration signal. The uncertainty estimation using the MGPRLF-PI algorithm, $\phi_{MGPRLF-PI}$, is represented as the following technique.

(22) $$\phi_{MGPRLF-PI}\left(k+1\right)=\phi_{MGPRLF-PI}\left(k\right)+\delta_{PI}\left(Y_{raw}\left(k\right)-Y_{MGPRLF-PI}\left(k\right)\right)$$

Here, $Y_{raw}\left(k\right)$ and $\delta_{PI}\;$ are the original raw signals that are collected by the vibration sensor and the coefficient, respectively. The MGPRLF-PI algorithm is a linear-based estimator. Apart from the simplicity of the MGPRLF-PI technique, robustness and reliability are two main limitations of this approach. To address the robustness, the Lyapunov algorithm is recommended in this research. The Lyapunov technique is integrated with the MGPRLF-PI observer to improve the robustness (hence is called MGPRLF-RPI). Thus, the Lyapunov function, $\upsilon_{\gamma }\left(e,X\left(k\right),\phi \left(k\right)\right)$, is denoted by the subsequent equivalence.

(23) $$\upsilon_{\gamma }\left(e,X\left(k\right),\phi \left(k\right)\right)={\mathbb{R}}_{\gamma }\left(e,X\left(k\right)\right)+\eta_{\gamma }\left(e\right)\phi \left(k\right)$$

Here, ${\mathbb{R}}_{\gamma }\left(e,X\left(k\right)\right)$ and $\eta_{\gamma }\left(e\right)\phi \left(k\right)$ are, respectively, the Hamilton–Jacobi discrimination and differentiable function of the uncertainty (unknown) condition. The Lyapunov procedure is robust and stable. Thus, the MGPRLF-RPI procedure is represented as the following definition.

(24) $$\{\begin{array}{c} X_{MGPRLF-RPI}\left(k+1\right)=[{\mathbb{C}}_{GPRLF}X_{MGPRLF-RPI}\left(k\right)+\delta_{i}X_{i}\left(k\right)+\delta_{o}Y_{MGPRLF-RPI}\left(k\right)\\ +\delta_{f}Y_{f}\left(k\right)]+e_{GPRLF}\left(k\right)+\phi_{MGPRLF-RPI}\\ Y_{MGPRLF-RPI}\left(k\right)={\left(\delta_{o}\right)}^{T}\left(x_{n}\right){\mathbb{C}}_{GPRLF}{}^{-1}\times X_{MGPRLF-RPI}\left(k\right) \end{array},$$

Here, $X_{MGPRLF-RPI}\left(k\right),Y_{MGPRLF-RPI}\left(k\right),$ and $\phi_{MGPRLF-RPI}$ are the state of the bearing signal estimation using the MGPRLF-RPI technique, the estimated signal by the MGPRLF-RPI method, and the uncertainty estimation using the MGPRLF-RPI algorithm, respectively. The uncertainty estimation using the MGPRLF-PI algorithm, $\phi_{MGPRLF-RPI}$, is represented as the following equation.

(25) $$\begin{array}{ll} \phi_{MGPRLF-RPI}\left(k+1\right) & =\phi_{MGPRLF-RPI}\left(k\right)+\delta_{RPI}\left(Y_{raw}\left(k\right)-Y_{MGPRLF-RPI}\left(k\right)\right)\\ & +\upsilon_{\gamma }\left(e_{MGPRLF},X_{MGPRLF-RPI}\left(k\right),\phi_{MGPRLF-RPI}\left(k\right)\right) \end{array}$$

Here, $\upsilon_{\gamma }\left(e_{MGPRLF},X_{MGPRLF-RPI}\left(k\right),\phi_{MGPRLF-RPI}\left(k\right)\right)$, and $\delta_{RPI}\;$ are the Lyapunov function to increase the robustness of the proposed algorithm and the coefficient, respectively. In this approach, the Lyapunov technique is selected to improve the robustness of signal estimation against the unknown condition in the vibration signal. The main challenge of nonlinear and non-stationary signals is uncertainty. To address this issue and increase the reliability in the MGPRLF-RPI, the combination of the adaptive technique and MGPRLF-RPI (MGPRLF-ARPI) that, henceforth, is called the adaptive digital twin (ADT) is recommended. The ADT procedure is signified using the following description.

(26) $$\{\begin{array}{c} X_{ADT}\left(k+1\right)=\left[{\mathbb{C}}_{GPRLF}X_{ADT}\left(k\right)+\delta_{i}X_{i}\left(k\right)+\delta_{o}Y_{ADT}\left(k\right)+\delta_{f}Y_{f}\left(k\right)\right]+\\ e_{GPRLF}\left(k\right)+\phi_{ADT}\\ Y_{ADT}\left(k\right)={\left(\delta_{o}\right)}^{T}\left(x_{n}\right){\mathbb{C}}_{GPRLF}{}^{-1}X_{ADT}\left(k\right) \end{array},$$

The uncertainty estimation using the ADT algorithm, $\phi_{ADT}$, is represented as the following equation.

(27) $$\begin{array}{ll} \phi_{ADT}\left(k+1\right)= & \phi_{ADT}\left(k\right)+\delta_{ADT-New}\left(Y_{raw}\left(k\right)-Y_{ADT}\left(k\right)\right)\\ & \;+\upsilon_{\gamma }\left(e_{MGPRLF},X_{ADT}\left(k\right),\phi_{ADT}\left(k\right)\right) \end{array}$$

Here, $\;X_{ADT}\left(k\right),Y_{ADT}\left(k\right),\phi_{ADT},\upsilon_{\gamma }\left(e_{MGPRLF},X_{ADT}\left(k\right),\phi_{ADT}\left(k\right)\right)$, and $\delta_{ADT-New}$ are the state of the bearing signal estimation using the proposed ADT technique, the estimated signal by the proposed ADT method, the uncertainty estimation using the proposed ADT algorithm, the effect of the Lyapunov function to improve the robustness in the proposed ADT algorithm, and the adaptive (update) coefficient for tuning the proposed ADT estimator, respectively. The adaptive approach is used to auto-tune the coefficient to estimate the unknown condition. The adaptive (update) coefficient, $\delta_{ADT-New}$, is calculated using the following definition.

(28) $$\delta_{ADT-New}=\delta_{RPI}\times \delta_{f}Y_{f}$$

### 3.2. Residual Signal Computation

Based on the previous section, the signals are modeled, and estimation is performed using the ADT technique. In this part, the residual signals are computed using the difference between the original raw signals, $Y_{raw}\left(k\right)$, and estimated raw signals using the proposed ADT algorithm, $Y_{ADT}\left(k\right)$. The residual signals array, $R_{ADT}\left(k\right)$, is computed using the following technique:

(29) $$R_{ADT}\left(k\right)=Y_{raw}\left(k\right)-Y_{ADT}\left(k\right)$$

Based on (27), the residual signal is a new feature that is more separable than the original signals. Thus, based on the power of signal estimation using adaptive digital twin, the normal and abnormal residual signals are distinguishable (hence is called fault detection). In addition, based on the above technique, abnormal signals in different types of faults allow for facile fault pattern recognition and crack size identification.

### 3.3. Signal Classification

To classify the residual signals, first, the residual signals are resampled, and the root means square (RMS) features are extracted from the resampled residual signals. The RMS resampled residual signal, $R_{ADT}{\left(k\right)}_{rms}$, is represented as the following equation.

(30) $$R_{ADT}{\left(k\right)}_{rms}=\sqrt{\frac{1}{T}\overset{T}{\underset{j=1}{\sum }}{\left(R_{ADT}\left(k\right)\right)}^{2}}$$

At this juncture, $R_{ADT}{\left(k\right)}_{rms}$ and *T* symbolize the resampled RMS value for the residual signal that is determined using the ADT technique, and the number of windows in this work, respectively. For the normal and each abnormal condition, the residual signals have 120,000 samples. Based on the induction motor and the CWRUBD conditions, the residual signal was segmented into 100 windows. Therefore, each window contains 1200 samples. To perform signal classification, the resampled RMS residual signals are determined for 100 windows. A support vector machine (SVM) is used for classification [27,30]. Additionally, 75% of the resampled RMS signals are used for training and 25% are selected for testing in the SVM. Table 3 shows the details of the training and testing dataset for the normal and abnormal conditions. Moreover, Table 4 illustrates the proposed algorithm steps for fault diagnosis of the bearing.

## 4. Experimental Result

**Figure 3:**
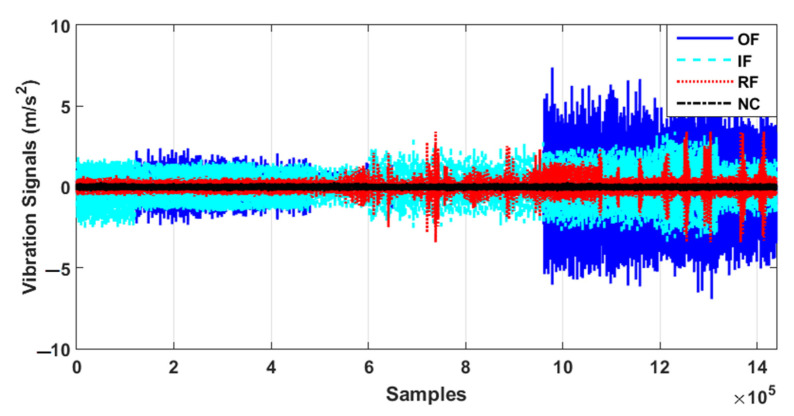
The original raw signals of the CWRUBD.

The CWRUBD is suggested to test the proposed algorithm. Figure 3 illustrates the original raw bearing signals in normal and abnormal conditions. Based on this figure, the classification using original signals is difficult and the accuracy of classification is very low. Regarding this figure, the signals in various conditions substantially overlap. The experimental results have three sub-parts: signal modeling and estimation using the ADT results, residual signal tests and results, and the classification results.

### 4.1. Signal Modeling and Estimation Using the ADT Results

**Figure 4:**
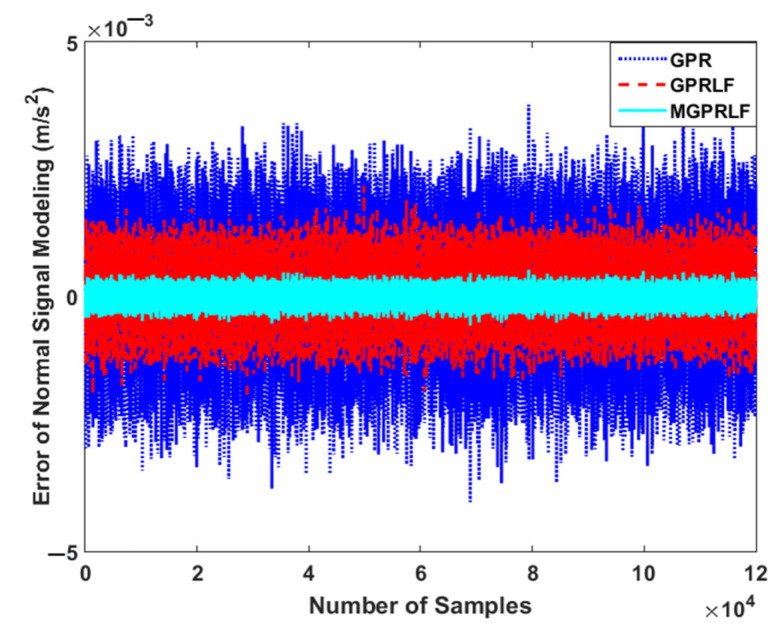
Error of system modeling using the proposed MGPRLF, GPRLF, and GPR algorithms.

To test the power of the proposed MGPRLF technique for vibration signal modeling in the normal condition, it is compared with the GPR and GPRLF techniques. Figure 4 shows the error of signal modeling to extract the state-space function from the original raw signal in the normal condition.

Based on Figure 4, the error of signal modeling for the proposed MGPRLF algorithm is less than the other two methods. This means the proposed MGPRLF technique is more robust and stable than the GPRLF and GPR techniques. The combination of the mathematical approach and data-driven technique increases the modeling resistance against uncertain conditions.

**Figure 5:**
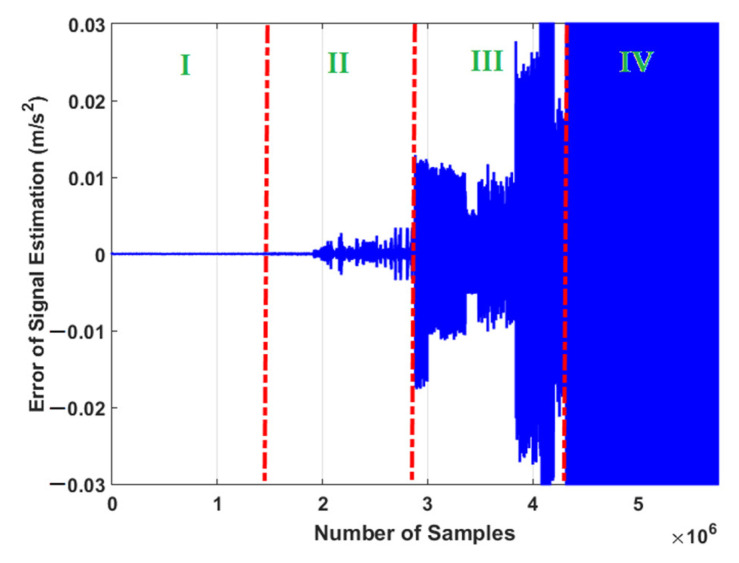
Error of signal estimation using proposed ADT algorithm: (I) NC, (II) RF, (III) IF, and (IV) OF.

The error of signal estimation using the proposed ADT is illustrated in Figure 5. Based on this figure, it is clear that the power of signal estimation for the normal condition (NC) is better than RF, IF, and OF. The reason for this level difference is that the modeling and estimation technique is tuned in the NC. This property of the estimation technique is used to amplify the difference of the error of the signal estimation in different conditions.

The reason for different error levels in each RF, IF, or OF region is the existence of different crack sizes.

### 4.2. Fault Pattern Recognition (Crack Identification)

**Figure 6:**
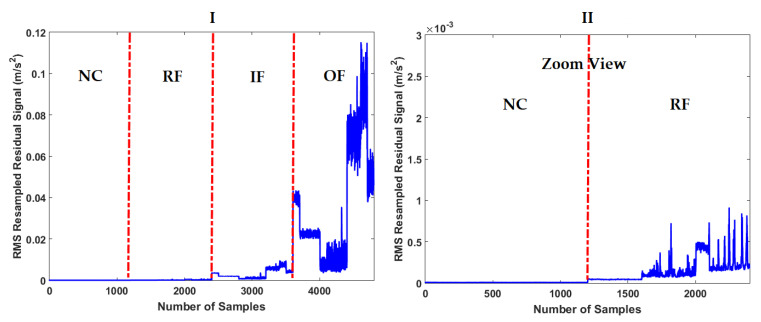
The RMS resampled residual signals using the proposed ADT algorithm: (I) for the NC, RF, IF, and OF and (II) zoom view.

**Figure 7:**
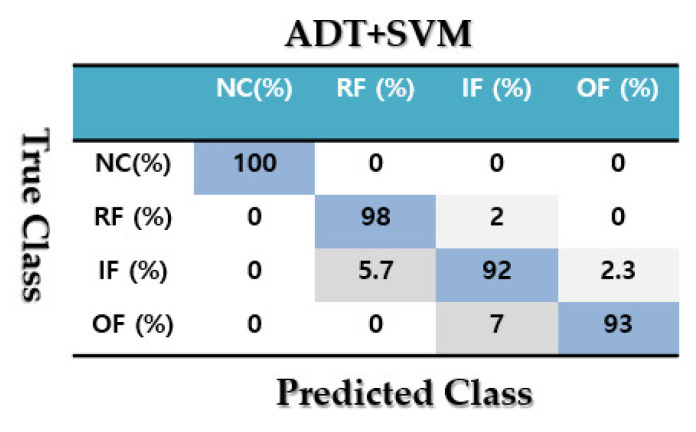
The average accuracies of crack identification using the proposed ADT+SVM scheme.

**Figure 8:**
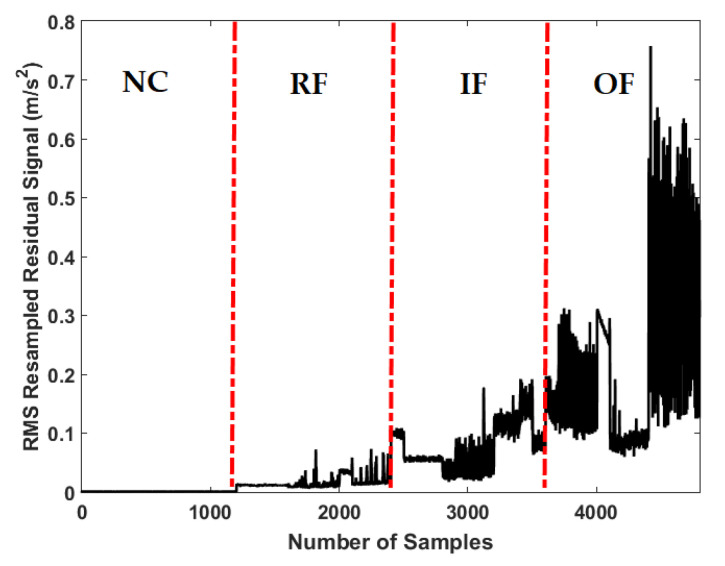
The RMS resampled residual signals using the MGPRLF-RPI technique for NC, RF, IF, and OF.

To test the fault pattern recognition and crack size identification using the CWRUBD, three techniques are compared in this part, the proposed ADT, the MGPRLF-RPI method, and the MGPRLF-PI approach. Figure 6, Figures 8 and 10 show the residual signals for the proposed ADT approach, the MGPRLF-RPI method, and the MGPRLF-PI technique, respectively. Based on Figure 3 and Figure 6, it is clear that the accuracy of the condition classification using the proposed ADT is better than that using the original RAW signal. Figure 7 demonstrates the confusion matrix to test the crack identification accuracy using the ADT + SVM. Based on Figure 7, the average accuracy for fault pattern recognition based on the proposed ADT+SVM is 95.75%. Moreover, Figure 8 shows the residual signals for the MGPRLF-RPI method. Based on the comparison of Figure 6 and Figure 8, the accuracy of fault pattern recognition (especially for IF and OF) using proposed ADT approach is better than MGPRLF-RPI method.

**Figure 9:**
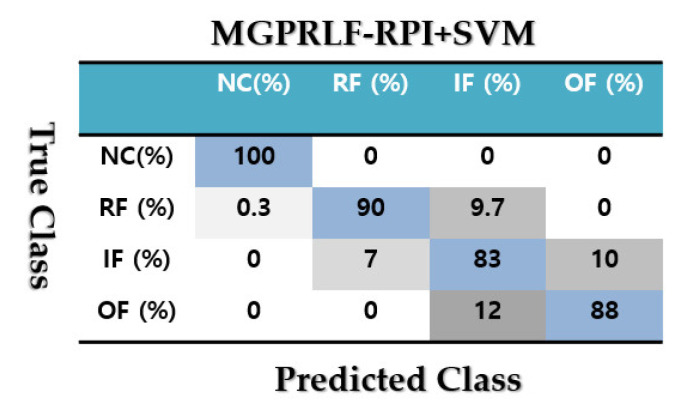
The average accuracies of crack identification using the MGPRLF-RPI+SVM technique.

Figure 9 demonstrates the confusion matrix to test the crack identification accuracy using the MGPRLF-RPI+SVM. Based on Figure 9, the average accuracy for fault pattern recognition based on the MGPRLF-RPI+SVM is 90.25%. Based on the comparison of Figure 7 and Figure 9, the accuracy of IF and OF fault pattern recognition using proposed ADT+SVM approach and the MGPRLF-RPI + SVM are 92%, 93%, 83%, and 88%, respectively. 

**Figure 10:**
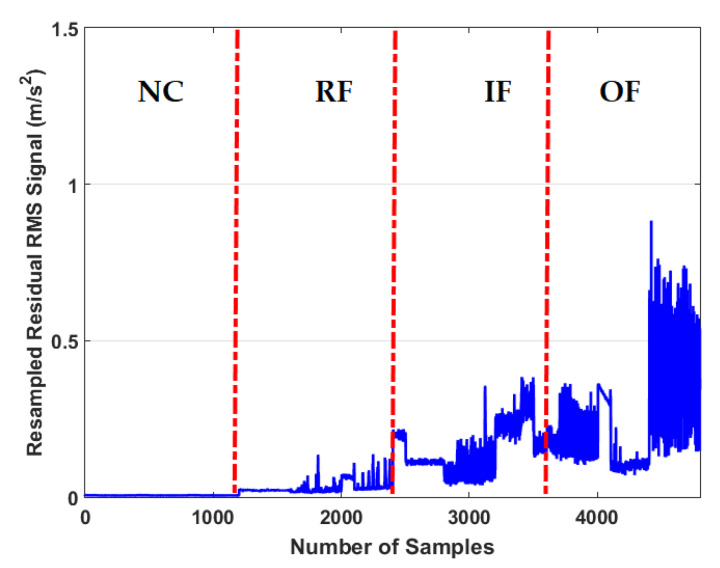
Resampled residual RMS signal using the MGPRLF-PI technique for the NC, RF, IF, and OF.

Figure 10 shows the residual signal for the MGPRLF-PI technique. Based on the comparison of Figure 6, Figure 8 and Figure 10, the accuracy of fault pattern recognition (especially for IF and OF) using proposed ADT approach is better than MGPRLF-RPI and MGPRLF-PI methods. 

**Figure 11:**
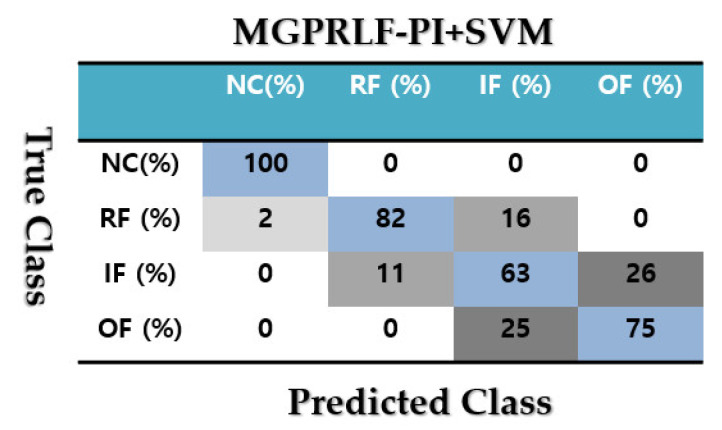
The average accuracies of crack identification using the MGPRLF-PI+SVM technique.

Figure 11 validates the confusion matrix to test the crack identification accuracy using the MGPRLF-PI + SVM. 

Based on the comparison of Figure 7, Figure 9 and Figure 11, the average sensitivities of the proposed ADT+SVM, MGPRLF-RPI+SVM, and MGPRLF-PI+SVM techniques are 95.75%, 90.25%, and 80%, respectively. Therefore, the proposed method has improved the crack identification by 5.5% compared to MGPRLF-RPI+SVM and 25.75% compared to MGPRLF-PI+SVM. The challenging areas in these figures are the overlap between the OF and IF residuals and the overlap between the RF and IF in some areas. Comparing Figure 6, Figure 7, Figure 8, Figure 9, Figure 10 and Figure 11, it can be seen that the proposed ADT algorithm, Figure 6 and Figure 7, has a lower overlap between conditions than the other two techniques shown in Figure 8, Figure 9, Figure 10 and Figure 11. The overlap and misclassification in the MGPRLF-PI technique, in Figure 10 and Figure 11, are higher than those using the MGPRLF-RPI method, in Figure 8 and Figure 9.

Based on the above figures, the main challenge using the MGPRLF-PI+SVM and MGPRLF-RPI+SVM techniques is the classification of inner and outer faults. As in MGPRLF-PI+SVM, the misclassifications of the inner and outer faults are 37% and 25%, respectively. Similarly, using the MGPRLF-RPI+SVM technique, this misclassification is reduced to about 17% for the inner fault and 12% for the outer fault, but in the proposed approach, the misclassification is reduced to about 8% for the inner and 7% for the outer mode. The crack identification accuracy is tested using the ADT + SVM, the MGPRLF-RPI + SVM, and the MGPRLF-PI + SVM. According to Table 3 of the crack identification section, the RMS resampled residual signals have 4800 samples, with 75% for training and 25% for testing, in the NC, RF, IF, and OF states. Table 5 demonstrates the average accuracy of the crack identification using the ADT + SVM, the MGPRLF-RPI + SVM, and the MGPRLF-PI + SVM techniques, respectively. To evaluate the robustness and reliability of the ADT + SVM, the MGPRLF-RPI + SVM, and the MGPRLF-PI + SVM techniques, 20 different tests were performed by changing the training and test data, and their average results are shown in the following table. Therefore, the proposed method has improved the crack identification by 5.5% compared to MGPRLF-RPI+SVM and 25.75% compared to MGPRLF-PI+SVM.

**Figure 12:**
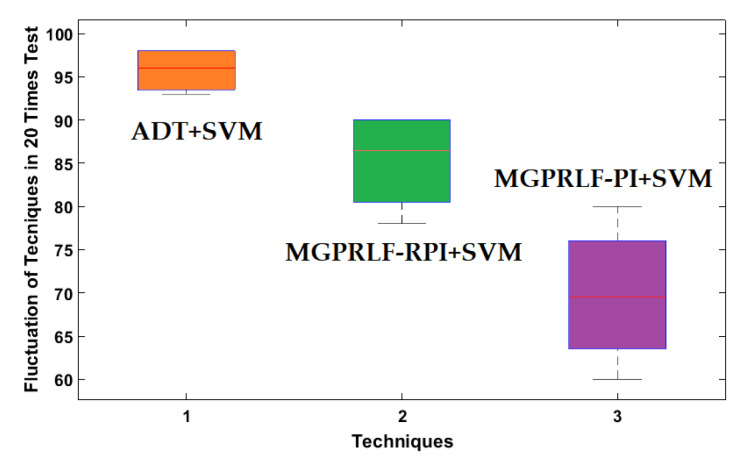
The average boxplots of crack identification fluctuation (NR, RF, IF, and OF) of the ADT+SVM, MGPRLF-RPI+SVM, and MGPRLF-PI+SVM techniques to test the reliability and robustness with 20 tests.

Figure 12 shows the repeatability and robustness of the three techniques when 20 different tests were performed by changing the training and test data. According to Figure 12, the amount of distortion using the proposed algorithm is less than the other two methods. This means that the proposed technique is more robust and reliable than the other two methods.

### 4.3. Crack Size Identification

**Figure 13:**
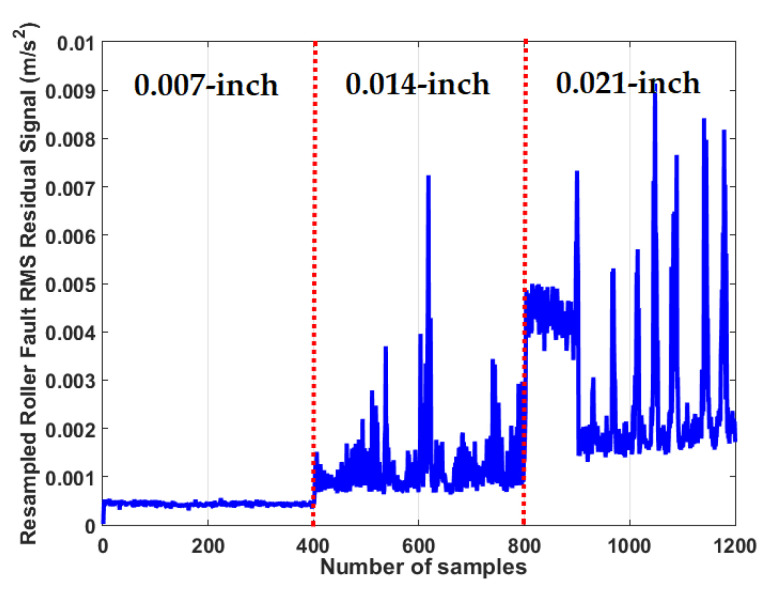
The RMS resampled roller fault residual signal using the proposed ADT algorithm for crack size identification.

**Figure 14:**
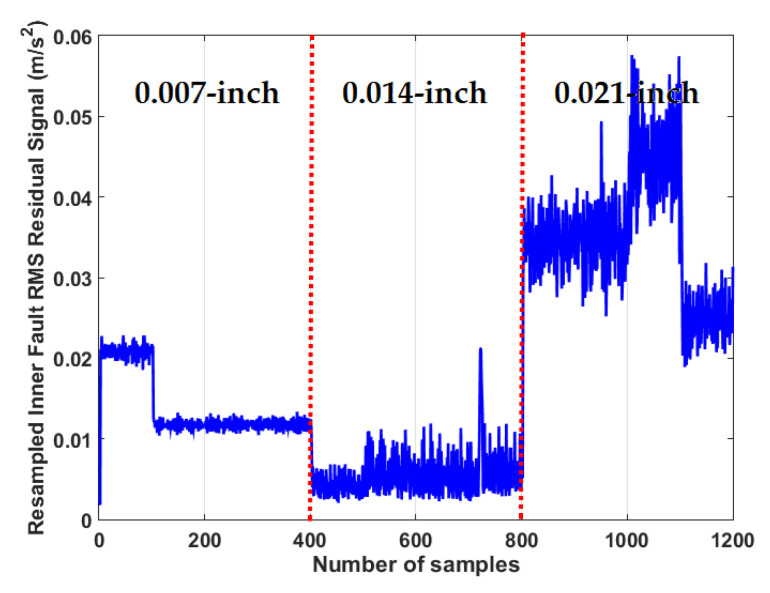
The RMS resampled inner fault residual signal using the proposed ADT algorithm for crack size identification.

**Figure 15:**
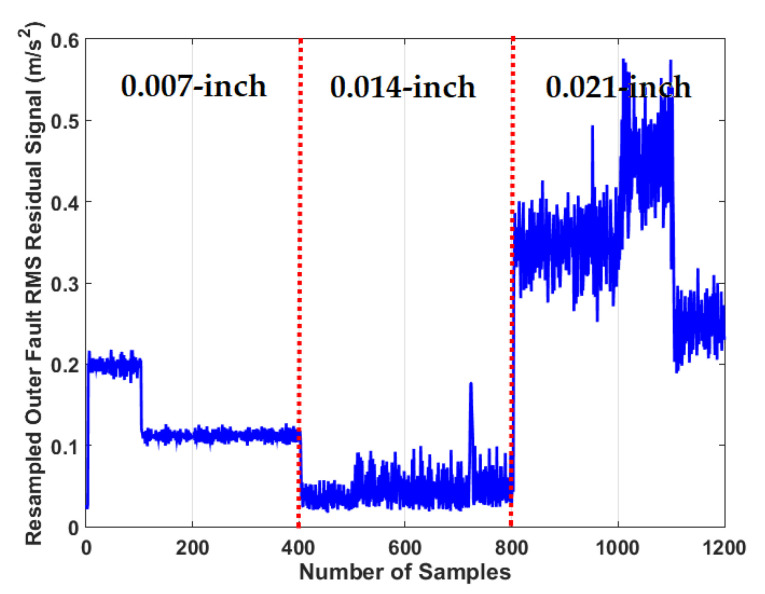
The RMS resampled outer fault residual signal using the proposed ADT algorithm for crack size identification.

To test the crack size identification using the CWRUBD, three techniques are compared in this part, the proposed ADT, the MGPRLF-RPI method, and the MGPRLF-PI approach. Figure 13, Figure 14 and Figure 15 show the roller, inner, and outer crack residual signals for the proposed ADT approach, respectively. Moreover, Table 6 demonstrates the identifications of the sizes of the cracks for the RF using the proposed ADT+SVM, MGPRLF-RPI+SVM, and MGPRLF-PI+SVM techniques, respectively. 

According to the above table, the average accuracy of classification by the suggested scheme (ADT+SVM) is better than those of the other two methods. Moreover, the proposed scheme has improved the size recognition for the RF by 8.33% and 16.66% compared to the MGPRLF-RPI+SVM and MGPRLF-PI+SVM techniques, respectively. In addition, Figure 14 illustrates the inner crack residual signal for the proposed ADT style. Table 7 shows the average accuracies of the inner fault size identification using the proposed ADT+SVM, MGPRLF-RPI+SVM, and MGPRLF-PI+SVM techniques. Furthermore, according to Table 7, the average accuracy of crack size classification by the proposed scheme (ADT+SVM) for the IF is better than the other two methods. Moreover, the proposed scheme has improved the size recognition of the IF by 9.66% and 16% compared to the MGPRLF-RPI+SVM and MGPRLF-PI+SVM techniques, respectively. 

Moreover, Figure 15 shows the outer crack residual signal for the proposed ADT scheme. The average accuracies of the outer crack size identification using the proposed ADT+SVM, MGPRLF-RPI+SVM, and MGPRLF-PI+SVM methods are illustrated in Table 8. Additionally, based on Table 8, the average accuracy of crack size classification is improved by the proposed scheme (ADT+SVM) for the OF. The ADT+SVM improved the size recognition for the OF by 9.33% and 16.33% compared to the MGPRLF-RPI+SVM, and MGPRLF-PI+SVM techniques, respectively. 

**Figure 16:**
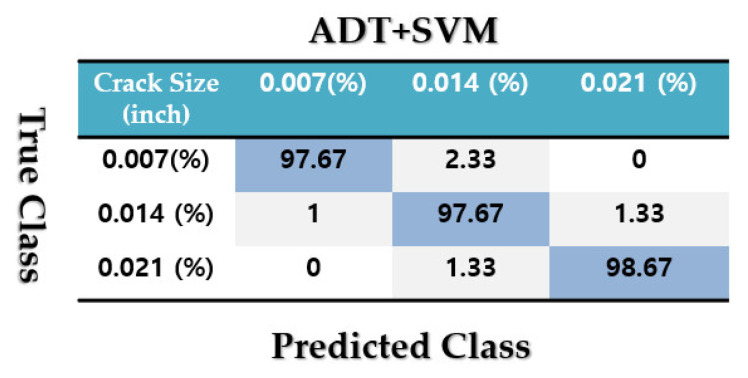
The average accuracies of crack size identification for the RF, IF, and OF using the proposed ADT+SVM scheme.

**Figure 17:**
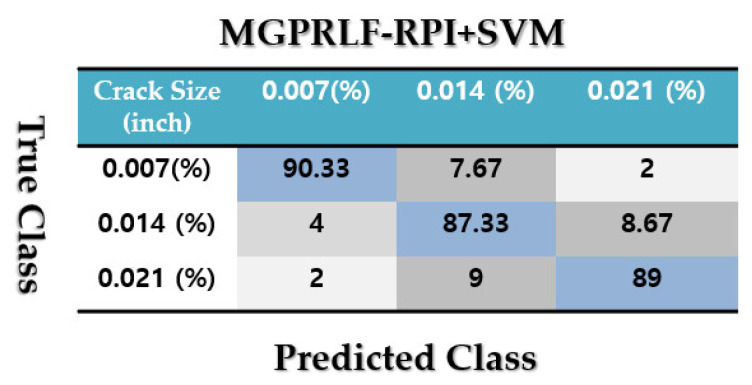
The average accuracies of crack size identification for the RF, IF, and OF using the proposed MGPRLF-RPI+SVM scheme.

**Figure 18:**
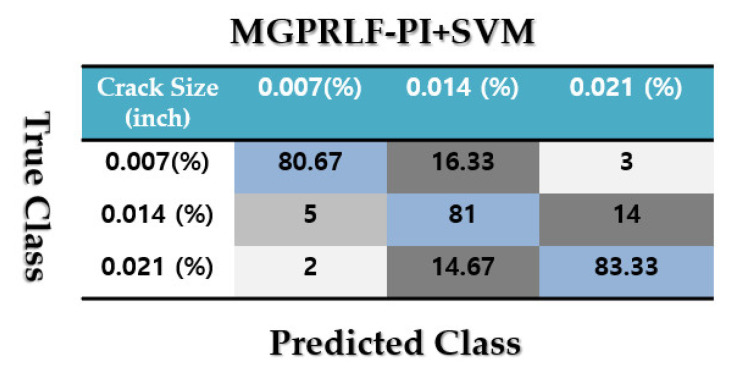
The average accuracies of crack size identification for the RF, IF, and OF using the proposed MGPRLF-PI+SVM scheme.

Figure 16, Figure 17 and Figure 18 display the confusion matrices to test the average crack size identification accuracies for the RF, IF, and OF using the ADT + SVM, the MGPRLF-RPI + SVM, and the MGPRLF-PI + SVM approaches, respectively. Based on these figures, the average crack size identification accuracies for the proposed ADT+SVM, MGPRLF-RPI+SVM, and MGPRLF-PI+SVM schemes are, respectively, 98%, 88.89%, and 81.67%. Therefore, the proposed ADT+SVM had improved the average accuracy of crack (RF, IF, and OF) size identification by 9.11% and 16.33% compared to the MGPRLF-RPI+SVM and MGPRLF-PI+SVM techniques, respectively. Regarding these figures, the misclassifications between 0.007-inch, 0.014-inch, and 0.021-inch cracks using the proposed ADT+SVM are lower than the other two techniques. 

**Figure 19:**
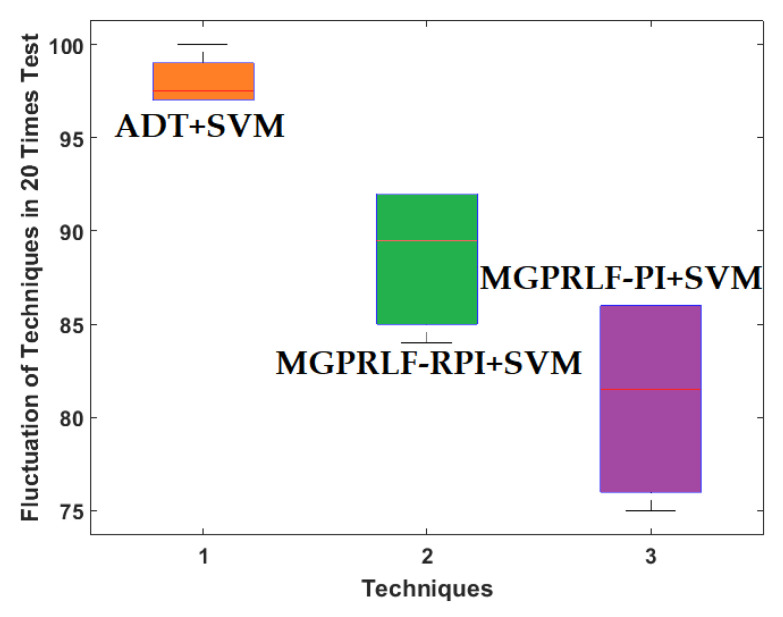
The average boxplots of crack size identification fluctuation (RF, IF, and OF) of the ADT+SVM, MGPRLF-RPI+SVM, and MGPRLF-PI+SVM to test the reliability and robustness of tests conducted 20 times.

Figure 19 shows the repeatability and robustness of the three techniques when 20 different tests were performed by changing the training and test data for the crack (RF, IF, and OF) size identification. According to Figure 19, the amount of distortion in the proposed algorithm is less than the other two approaches. Thus, the proposed ADT+SVM is more robust and reliable than the other two procedures. 

## 5. Conclusions

In this work, the adaptive digital twin (ADT) was recommended for the bearing fault diagnosis and crack size identification tasks. Two steps were performed to design the ADT. In the first step, the normal signals collected by the vibration sensors were modeled, and their state-space function was extracted. To do this, a combination of mathematical and data-driven methods is proposed. The core of the data-driven method was the GPR algorithm, which had its robustness and accuracy of signal modeling improved in two stages by Laguerre algorithms and the fuzzy logic algorithm, respectively. 

In the second step, an estimator is designed for the normal signal and, after tuning, it is tested for all signals. The main principles of the proposed estimator are based on the combination of the proposed modeling technique and the PI observer. Lyapunov and adaptive algorithms were proposed in this work to strengthen the resistance and increase the reliability of the digital twin. After designing the proposed ADT to strengthen the power of fault classification, two steps were performed. First, the residual signal, which is the result of the difference between the original and estimated signals, is calculated. Then, the residual signals are resampled, and the RMS features are extracted. Next, the new signals were sent to check the classification accuracy by the SVM algorithm in two stages: fault diagnosis and crack size identification. The proposed technique was tested using the CWRUBD. In general, the classification accuracy of the proposed scheme (ADT+SVM) is 97.5%, which improved the accuracy of MGPRLF-RPI+SVM and MGPRLF-PI+SVM by 8.2% and 18.7%, respectively. The simplicity, reliability, and high accuracy in modeling are the main advantages of this adaptive technique. To improve the classification accuracy for multi-crack faults, in future research, our goal is to improve the performance response of this algorithm by combining machine/deep learning and observation techniques. Thus, it will be possible to improve the classification by designing noise reduction in the preprocessing section.

## Figures and Tables

**Table 1:** The CWRUBD experimental information.

Information	Detail
Power of the induction motor	2 [hp]
Bearing rotating speeds	1730 [RPM]; 1750 [RPM]; 1772 [RPM]; 1797 [RPM]
Crack sizes	0.007 [inches]; 0.014 [inches]; 0.021 [inch]
Sampling rate frequency	48 [KHz]
Type of bearing	6205-2RS JEM SKF
Number of rollers	9
Roller’s stiffness	$5.96\times 10^{7}\;\left(\frac{N}{m}\right)$
Outer’s stiffness	$1.31\times 10^{5}\;\left(\frac{N}{m}\right)$
Shaft’s stiffness	$23.3\times 10^{6}\;\left(\frac{N}{m}\right)$
Outer’s Mass	$2.7\;\left(\mathrm{Kg}\right)$
Shaft’s Mass	$1.36\;\left(\mathrm{Kg}\right)$
Defect depth	$2\;\left(\mathrm{mm}\right)$
Pitch diameter	$39.04\;\left(\mathrm{mm}\right)$
Roller diameter	$7.940\;\left(\mathrm{mm}\right)$

**Table 2:** The CWRUBD experimental information.

Classes	Motor Torque Load [hp]	Crack Sizes [inch]
NC	0,1,2,3	-
RF	0,1,2,3	0.007; 0.014; 0.021
IF	0,1,2,3	0.007; 0.014; 0.021
OF	0,1,2,3	0.007; 0.014; 0.021

**Table 3:** Information regarding the training and testing for classification using the SVM.

Conditions	Number of Training Samples	Number of Testing Samples
Crack Identification		
NC	900	300
RF	900	300
IF	900	300
OF	900	300
Size Identification for RF		
0.007-inch	300	100
0.014-inch	300	100
0.021-inch	300	100
Size Identification for IF		
0.007-inch	300	100
0.014-inch	300	100
0.021-inch	300	100
Size Identification for OF		
0.007-inch	300	100
0.014-inch	300	100
0.021-inch	300	100

**Table 4:** The proposed algorithm uses a combination of the adaptive digital twin (ADT) and SVM for fault diagnosis of the bearing.

1:	**Adaptive Digital Twin Design** Implement the state-space GPR algorithm, Equation (1).
2:	Improve the robustness of autoregressive technique by combining autoregressive algorithm with the Laguerre filter. Equations (3) and (4)
3:	Improve the accuracy and flexibility of GPRL using the GPRLF algorithm, Equation (9).
4:	Mathematical modeling of the bearing, Equation (19).
5:	Improve the performance of modeling in the digital twin using the MGPRLF algorithm, Equation (20).
6:	Implement the combination of PI observer and MGPRLF for signal estimation, Equations (21) and (22).
7:	Improve the robustness of MGPRLF-PI for signal estimation using MGPRLF-RPI, Equations (24) and (25).
8:	signal estimation using the proposed ADT, Equations (26) and (27).
9:	**Residual Signal Computation** Compute the residual signals, Equation (29).
	**Residual Signals Fault Classification**
10:	Compute the resampled RMS residual signals, Equation (30).
11:	Perform classification of the resampled RMS residual signals using the SVM [20,23].

**Table 5:** The average accuracies of crack identification using ADT+SVM, MGPRLF-RPI+SVM, and MGPRLF-PI+SVM techniques.

Classes	ADT +SVM (%)	MGPRLF-RPI +SVM (%)	MGPRLF-RPI +SVM (%)
NC	100	100	100
RF	98	90	82
IF	92	83	63
OF	93	88	75
Average	95.75	90.25	80

**Table 6:** The average accuracies of the roller crack size identification using the ADT+SVM, MGPRLF-RPI+SVM, and MGPRLF-PI+SVM techniques.

Size (inch)	ADT +SVM (%)	MGPRLF-RPI +SVM (%)	MGPRLF-RPI +SVM (%)
0.007	98	90	80
0.014	96	88	82
0.021	98	89	80
Average	97.33	89	80.67

**Table 7:** The average accuracies of the inner crack size identification using the ADT+SVM, MGPRLF-RPI+SVM, and MGPRLF-PI+SVM techniques.

Size (inch)	ADT +SVM (%)	MGPRLF-RPI +SVM (%)	MGPRLF-RPI +SVM (%)
0.007	98	90	82
0.014	98	86	81
0.021	99	90	84
Average	98.33	88.67	82.33

**Table 8:** The average accuracies of outer crack size identification using the ADT+SVM, MGPRLF-RPI+SVM, and MGPRLF-PI+SVM techniques.

Size (inch)	ADT +SVM (%)	MGPRLF-RPI +SVM (%)	MGPRLF-RPI +SVM (%)
0.007	97	91	80
0.014	99	88	80
0.021	99	88	86
Average	98.33	89	82

## Data Availability

The data are publicly available.

## Nomenclature

ADT	Adaptive Digital Twin
SVM	Support Vector Machine
GPR	Gaussian Process Regression
PI	Proportional Integral
NC	Normal Condition
IF	Inner Fault
hp	horsepower
GPRLF	The combination of GPRL and fuzzy approach
MGPRLF-PI	PI observer with MGPRLF modeling
MGPRLF-ARPI (ADT)	The combination of MGPRLF-RPI observer and adaptive approach that is called adaptive digital twin
$X_{i}\left(k\right)$	The measurable vibration signal
$Y_{GPR}\left(k\right)$	The signal modeled by the GPR technique
$(\delta_{i},\;\left(\left(\delta_{o}{)}^{T}\left(x_{n}\right)\right)\right)$	The coefficient of signal modeling using the GPR algorithm
α	Signal variance
k	Kernel width
$Y_{GPRL}\left(k\right)$	The modeled signal by the GPRL method
CoG	Center of Gravity
$e_{GPRLF}\left(k\right)$	The error of signal modeling using the GPRLF algorithm
$Y_{f}\left(k\right)$	The modeled signal using the fuzzy algorithm to improve the accuracy and flexibility
$\delta_{f}$	The coefficient of the modeled signal using the fuzzy algorithm
${\mathbb{Z}}_{D\left(q\right)}$	The mass of bearing matrices
${\mathbb{N}}_{D}\left(q,\dot{q}\right)$	A nonlinear term for modeling the bearing
$\theta_{RF}$	The effect of the roller fault
$\theta_{OF}$	The effect of the outer fault
	The number of rollers in the bearing
$\delta_{XD}\left(X_{D}\left(k\right),X_{Di}\left(k\right)\right)$	The nonlinear term of the bearing using mathematically based vibration modeling
$X_{M}$	The state of the vibration signal modeling using the mathematical approach
$\delta_{YD}$	The coefficient
$Y_{GPRLF}\left(k\right)$	The modeled signal by the GPRLF method
$X_{MGPRLF-PI}\left(k\right)$	The state of the bearing signal estimation using the MGPRLF-PI technique
$Y_{raw}\left(k\right)$	The original raw signals that are collected by the vibration sensor
$\upsilon_{\gamma }\left(e,X\left(k\right),\phi \left(k\right)\right)$	The Lyapunov function
$\eta_{\gamma }\left(e\right)\phi \left(k\right)$	Differentiable function of the uncertainty (unknown) condition
$X_{MGPRLF-RPI}\left(k\right)$	The state of the bearing signal estimation using the MGPRLF-RPI technique
$\upsilon_{\gamma }\left(e_{MGPRLF},X_{MGPRLF-RPI}\left(k\right),\phi_{MGPRLF-RPI}\left(k\right)\right)$	The Lyapunov function to increase the robustness of the proposed algorithm
$\;X_{ADT}\left(k\right)$	The state of the bearing signal estimation using the proposed ADT technique
$\phi_{ADT}$	The uncertainty estimation using the proposed ADT algorithm
$\delta_{ADT-New}$	The adaptive (update) coefficient for tuning the proposed ADT estimator
$R_{ADT}\left(k\right)$	Residual signal using proposed ADT method
ADT + SVM	The combination of ADT and SVM
MGPRLF-PI + SVM	The combination of MGPRLF-PI and SVM
RMS	Root Means Square
CWRUBD	Case Western Reserve University Bearing Dataset
FIE	Fuzzy Inference Engine
RPM	Rotation Per Minute
RF	Roller Fault
OF	Outer Fault
GPRL	The combination of GPR and Laguerre technique
MGPRLF	The combination of mathematical modeling and GPRLF
MGPRLF-RPI	The combination of MGPRLF-PI observer and Lyapunov approach
$X_{GPR}\left(k\right)$	The state of the bearing signal modeling using the GPR technique
$e_{GPR}\left(k\right)$	The error of signal modeling using the GPR algorithm
${\mathbb{C}}_{GPR}$	The covariance matrix using the GPR technique
ε	Noise variance
$e_{GPRL}\left(k\right)$	The error of signal modeling using the GPRL algorithm
$X_{GPRL}\left(k\right)$	State of the bearing signal modeling using the GPRL technique
${\mathbb{C}}_{GPRL}$	The covariance matrix using the GPRL algorithm
$X_{GPRLF}\left(k\right)$	The state of the bearing signal modeling using the GPRLF technique
$Y_{GPRLF}\left(k\right)$	The modeled signal by the GPRLF method
${\mathbb{C}}_{GPRLF}$	The covariance matrix using the GPRLF algorithm
$F_{D\left(q\right)}$	The external source forces
$\ddot{q}$	The acceleration vibration signal that is measured by a vibration sensor
$\theta_{D}$	And the unknown condition (hence is called uncertainty)
$\theta_{IF}$	The effect of the inner fault
$\varphi_{\alpha }$	The angular velocity of rotor
$\theta_{f}$	The difference between two reference angular positions
$\chi_{XD}\left(X_{D}\left(k\right),X_{Di}\left(k\right)\right)$	The uncertainty term of the bearing using mathematically based vibration modeling
$Y_{M}\left(k\right)$	The modeled vibration signal using the mathematical technique
$X_{GPRLF}\left(k\right)$	The state of the bearing signal modeling using the MGPRLF technique
$Y_{MGPRLF-PI}\left(k\right)$	The estimated signal by the MGPRLF-PI method
$\phi_{MGPRLF-PI}$	The uncertainty estimation using the MGPRLF-PI algorithm
$\delta_{PI}$	The coefficient of PI observer
${\mathbb{R}}_{\gamma }\left(e,X\left(k\right)\right)$	The Hamilton–Jacobi discrimination
$Y_{MGPRLF-RPI}\left(k\right)$	The estimated signal by the MGPRLF-RPI method
$\phi_{MGPRLF-RPI}$	The uncertainty estimation using the MGPRLF-RPI algorithm
$\delta_{RPI}$	The coefficient of the RPI technique
$Y_{ADT}\left(k\right)$	The estimated signal by the proposed ADT method
$\upsilon_{\gamma }\left(e_{MGPRLF},X_{ADT}\left(k\right),\phi_{ADT}\left(k\right)\right)$	The effect of the Lyapunov function to improve the robustness in the proposed ADT algorithm
$R_{ADT}{\left(k\right)}_{rms}$	The RMS resampled residual signal using proposed ADT
*T*	The number of windows
MGPRLF-RPI + SVM	The combination of MGPRLF-RPI and SVM
